# Supplementary material for: Infertile men with semen parameters above WHO reference limits at first assessment may deserve a second semen analysis: Challenging the guidelines in the real-life scenario
Source: PLoS One. 2023 Jan 19;18(1):e0280519. doi: 10.1371/journal.pone.0280519 (PMC9851544; doi:10.1371/journal.pone.0280519)
Supplement: Attachment — Submitted filename: Response to Reviewers - 14 07 2022.doc [file pone.0280519.s002.doc]

**Dr. Emily Chenette, PhD**

**Editor-in-Chief,** PLOS ONE

Dr. Stefan Schlatt

Academic Editor, PLOS ONE

Milan, July 14^th^, 2022

Dear Dr Chenette,

dear Dr. Schlatt,

please find enclosed the revised version of the manuscript titled “**Infertile men with normal semen parameters at first assessment may deserve a second semen analysis: challenging the guidelines in the real-life scenario**” (PONE-D-22-12621- Authors: Luca Boeri, et al.) to be considered for publication in PLOS ONE.

We are very grateful to the Reviewers for their insightful comments to our paper.

List of the changes made in the manuscript:

**REVIEWER #1**

**COMMENT#1.**

There is no comprehensive description of how the basic semen analysis was performed. Data on variability and compliance with e.g. demand for comparison of replicate assessments, use of equipment able to provide reliable results, internal and external quality control is essential for this study.

**A1.** We thank the Reviewer#1 for this insightful comment. We have included a detailed description of basic semen analysis and quality control methods in the Methods section of the manuscript.

**COMMENT#2.**

Furthermore, although it is true that there is definition of infertility based on TTP=< 12 months, but this has been challenged not the least based on older comprehensive studies (Tietze 1968 e.g.) and recent studied showing huge differences between short time to pregnancy and WHO "reference limits" - and those "reference limits" have also been challenged in the most recent WHO manual (2022).

**A2**. We thank the Reviewer#1 for this comment. It is well known that infertility is not related to the mere sperm quality, but we relied on the WHO definition of infertility, which is the one used by most of the international scientific societies (e.g., European Association of Urology Guidelines; American Society for Reproductive Medicine).

**COMMENT#3.**

Authors claim semen assessments were done "within two hours". This is clearly NOT in compliance with WHO 2010: page 21, 2.5 Sperm Motility second paragraph: "Sperm motility within semen should be assessed as soon as possible after liquefaction of the sample, preferably at 30 minutes, but in any case within 1 hour, following ejaculation, to limit the deleterious effects of dehydration, pH or changes in temperature on motily."

**A3**. We thank the Reviewer#1 for this critical comment. During the revision of our manuscript, we have double checked once more the whole procedure with our laboratory biologists and indeed we may confirm that all samples have been and are rigorously analysed within 60 min. from collection. The text has been revised accordingly.

**COMMENT#4.**

This calls in question to what extent reliable laboratory methods have been used. Lack of compliance with WHO recommendations can indicate that the assessments are influenced by significant technical variability - and that the reported change from "normal" to "abnormal" to a significant degree depend on technical /laboratory/ errors.

**A4**. As comprehensively detailed before, our laboratory analyzed all semen samples strictly according to WHO indications. We have also included internal and external quality control throughout the Methods section of the manuscript.

We hope that the paper is now suitable to be considered for publication in the Original Articles section of PLOS ONE*.*

Sincerely yours,

Andrea Salonia, on behalf of all the authors

Andrea Salonia, MD, PhD, FECSM

University Vita-Salute San Raffaele

Division of Experimental Oncology/Unit of Urology, URI-Urological Research Institute

IRCCS Ospedale San Raffaele

Email: [salonia.andrea@hsr.it](mailto:salonia.andrea@hsr.it)
