# Supplementary material for: Infertile men with semen parameters above WHO reference limits at first assessment may deserve a second semen analysis: Challenging the guidelines in the real-life scenario
Source: PLoS One. 2023 Jan 19;18(1):e0280519. doi: 10.1371/journal.pone.0280519 (PMC9851544; doi:10.1371/journal.pone.0280519)
Supplement: Attachment — Submitted filename: Response to Reviewers - Sept 18 2022.doc [file pone.0280519.s003.doc]

**Dr. Emily Chenette, PhD**

Editor-in-Chief**,** PLOS ONE

**Dr. Stefan Schlatt**

Academic Editor, PLOS ONE

Milan, September 18^th^, 2022

Dear Dr Chenette,

dear Dr. Schlatt,

please find enclosed the revised version of the manuscript titled “**Infertile men with normal semen parameters at first assessment may deserve a second semen analysis: challenging the guidelines in the real-life scenario**” (PONE-D-22-12621R1- Authors: Luca Boeri, et al.) to be considered for publication in PLOS ONE.

We are very grateful to the Reviewers for their insightful comments to our paper.

List of the changes made in the manuscript:

**Journal Requirements**

Please review your reference list to ensure that it is complete and correct. If you have cited papers that have been retracted, please include the rationale for doing so in the manuscript text, or remove these references and replace them with relevant current references. Any changes to the reference list should be mentioned in the rebuttal letter that accompanies your revised manuscript. If you need to cite a retracted article, indicate the article’s retracted status in the References list and also include a citation and full reference for the retraction notice.

**A1.** The whole Reference list has been reviewed carefully; no papers have been retracted.

**REVIEWER #1**

**COMMENT#1.**

Authors only work with sperm concentration, but declares sperm numbers were calculated. It was clear already 2010 that the WHO manual recommends sperm number rather than concentration that depends on both sperm number and dilution by fluid from other sources.

**A1.** We thank the Reviewer#1 for this critical comment. Accordingly, total sperm number data has been included in the Results section of the manuscript. Thus, we have included total sperm number instead of sperm concentration in the analyses and previous findings were confirmed. The text has been revised accordingly.

**COMMENT#2.**

Variation in ejaculatory abstinence time can be an important source for variation. There is not even a discussion about this and no data available, only in M&M that abstinence time was 2-7 days. Could there be individual variability due to variation in abstinence time (or even ejaculatory frequency) before the two semen analyses?

**A2**. Ejaculatory abstinence time was similar between the two semen analyses [Median (IQR) 3 (1-4) days]. The Results section of the manuscript has been revised, accordingly.

**COMMENT#3.**

Have the authors considered that limits above the lower 5th percentile "limit" byt the WHO 2010 and 2021 could be better to distinguish between men with semen abnormalities and men without? Looking at e.g. Keihani et al 2021 indicating that men in couples with short time to initiation of pregnancy have much higher semen analysis results compared to men in couples with 5-12 months. Thus, I do agree with the authors that the so called WHO limits (which should not be mistaken for limits between fertile and subfertile men) do mean that there is a risk to miss men with fertility problems. What I miss (or have missed) is a discussion whether higher DECISION LIMITS could be an alternative solution to repeating semen analysis

**A3**. We thank the Reviewer#1 for this insightful comment. We agree with the Reviewer#1 and the WHO2021 suggesting that semen parameters per se cannot distinguish between fertile vs. infertile men. However, here we included infertile men only as for WHO definition (i.e., not conceiving after at least 12 months of unprotected intercourses regardless of whether or not a pregnancy ultimately occurs) which is actually independent from semen analysis parameters. Thus, semen parameters are used in clinical practice to guide diagnosis testing and the choice of treatment options for infertile men. Therefore, we aimed to challenge international guidelines concerning the need for a single or confirmative semen analysis. The need to use decision limits in clinical practice has been included in the Discussion section of the manuscript.

We hope that the paper is now suitable to be considered for publication in the Original Articles section of PLOS ONE*.*

Sincerely yours,

Andrea Salonia, on behalf of all the authors

Andrea Salonia, MD, PhD, FECSM

Vita-Salute San Raffaele University

Division of Experimental Oncology/Unit of Urology, URI-Urological Research Institute

IRCCS Ospedale San Raffaele

Email: [salonia.andrea@hsr.it](mailto:salonia.andrea@hsr.it)
