# Supplementary material for: Infertile men with semen parameters above WHO reference limits at first assessment may deserve a second semen analysis: Challenging the guidelines in the real-life scenario
Source: PLoS One. 2023 Jan 19;18(1):e0280519. doi: 10.1371/journal.pone.0280519 (PMC9851544; doi:10.1371/journal.pone.0280519)
Supplement: Attachment — Submitted filename: Response to Reviewers - November 06 2022.doc [file pone.0280519.s004.doc]

**Dr. Emily Chenette, PhD**

Editor-in-Chief**,** PLOS ONE

**Dr. Stefan Schlatt**

Academic Editor, PLOS ONE

Milan, November 06^th^, 2022

Dear Dr Chenette,

dear Dr. Schlatt,

please find enclosed the revised version of the manuscript titled “**Infertile men with semen parameters above WHO reference limits at first assessment may deserve a second semen analysis: challenging the guidelines in the real-life scenario**” (PONE-D-22-12621R2- Authors: Luca Boeri, et al.) to be considered for publication in PLOS ONE.

We are very grateful to the Reviewers for their insightful comments to our paper.

List of the changes made in the manuscript:

**REVIEWER #1**

**COMMENT#1.**

The supporting information is not sufficiently transparent.

a. Many labels and contents are in Italian - must be in English

b. Many cells have only an Error indication

c. A complete lack of explanations of column labels, how the reader should be able to analyse the data (e.g. compare first and second sample).

**A1.** We thank the Reviewer#1 for this comment. The supporting information file has been revised.

**COMMENT#2.**

The systematic use of terms like "normal semen" and "abnormal semen" although even WHO has pointed out that data should not be interpreted in that way: reference limits in WHO 2010 and 2021 are NOT limits between fertility and infertility. It would be better to refer to "men with semen results below/above WHO reference limts"

WHO 2021 even points to the fact that male factor infertility have different causes, and therefore it is not very likely that there exists ONE true limit concerning semen analysis results for all causes - decision limits are more likely to support the clinical work with male reproductive disorders.

**A2**. We thank the Reviewer#1 for this comment. The entire text has been revised accordingly.

**COMMENT#3.**

In table one, authors show the range of a number semen analysis parameters. It is strange that among men with "normal" first and second sample, low sperm numbers, concentrations, volumes etc are found!

**A3**. Table 1 reports baseline descriptive statistics of men according to their second semen analysis. In details Group 1 includes men with two consecutive semen analyses above WHO limits and Group 2 includes infertile men with a first semen sample above and a consecutive second one below limits. Of note, sperm parameters included in table 1 are baseline values (meaning at first assessment). The primary aim of table 1 is showing baseline characteristics of men to identify potential discrepancies within the two groups. Moreover, sperm concentration and volume were note lower in Group 1 compared to group 2.

**COMMENT#4.**

Concerning the abstinence time - there are only results on group level available. What would be of interest is the comparison of each individuals abstinence time for the two samples (average difference and variability).

**A4**. We thank the Reviewer#1 for this comment. We performed a Wilcoxon Signed Rank Test to assess potential difference in each individual abstinence time for the two samples. No statistical difference was noted. Difference in abstinence time for Group 1: average 0.32 days; variability 0.2; difference in abstinence time for Group 2: average 0.33 days; variability 0.2. The text has been revised accordingly.

**COMMENT#5.**

A very interesting aspect that has not been approached (as I can see it) is that the WHO reference limits 2010 and 2021 are too low! Againg thinking about e.g. Keihani 2021 this is not unlikely at all. The data the authors have could carry important information about this: could it be that using higher cut-offs would make a majority of men with first "normal" result to be above the WHO 2010/2021 level but under a tentative, higher cut-off? A graph showing the results of "normal-abnormal" men's first results in relation to cut-off level and compared to "normal-normal" men's

Of course, this would mean that the heavily promoted need to for a second semen analysis based on the often misunderstood WHO 2010/2021 reference limits becomes of less interest. But the matter deserves serious scientific attention not the least since the WHO 2021 points to the weaknesses of the presented reference limits.

**A5**. We thank the Reviewer#1 for this comment. We totally agree that reference limits should be taken with caution in clinical practice. Particularly due to the fact that they derived from the 5^th^ percentile of distribution from a cohort of fertile men. However, the WHO reference criteria are commonly used in clinical practice and the proposal of new cut-offs are beyond the aim of our study. In this study we aimed to identify baseline characteristics of men with semen parameters above the WHO limits (currently used worldwide) to dictate a second test. We believe that our study has a strong implication and relevance in the everyday clinical practice. We will conduct a new study in the near future thanks to your suggestion to depict different scenarios with higher limits.

**COMMENT#6.**

Authors repeatevly use the term"infertile men", but the definition is not sufficiently described. How where female factors ruled out?

**A6**. As reported in the Methods section, couple’s infertility was defined by WHO definition (not conceiving a pregnancy after at least 12 months of unprotected intercourses regardless of whether or not a pregnancy ultimately occurs). Moreover, each men underwent a comprehensive clinical, physical, hormonal, genetic and seminal investigation to identify the cause of male infertility. Male factor infertility was defined after a comprehensive diagnostic evaluation of all the female partners by expert Gynaecologists, which included a detailed medical, reproductive and family history as well as a general and gynaecological physical examination. Furthermore, the ovulatory status, ovarian reserve testing, the structure and patency of the female reproductive tract were requested in all cases. The text has been revised accordingly.

We hope that the paper is now suitable to be considered for publication in the Original Articles section of PLOS ONE*.*

Sincerely yours,

Andrea Salonia, on behalf of all the authors

Andrea Salonia, MD, PhD, FECSM

Vita-Salute San Raffaele University

Division of Experimental Oncology/Unit of Urology, URI-Urological Research Institute

IRCCS Ospedale San Raffaele

Email: [salonia.andrea@hsr.it](mailto:salonia.andrea@hsr.it)
